# Client, provider, and visit factors associated with quality in contraceptive counseling in Mexico: an exploratory cross-sectional analysis

**DOI:** 10.1186/s12978-021-01291-9

**Published:** 2021-12-09

**Authors:** Kay Walker, Ndola Prata, Maureen Lahiff, Ximena Quintero, Kelsey Holt

**Affiliations:** 1 School of Public Health, University of California, Berkeley, CA USA; 2 School of Medicine, University of California, San Francisco, CA USA; 3 Bixby Center for Population, Health and Sustainability, University of California, Berkeley, CA USA; 4 Mexican Family Planning Association, Mexico City, Mexico

**Keywords:** Contraceptive counseling, Contraceptives, Disrespect and abuse, Family planning, Human rights, Mexico, Post-partum, Quality of care, Youth

## Abstract

**Background:**

Monitoring clients’ experiences with contraceptive care is vital to inform quality improvement efforts and ensure fulfillment of individuals’ human rights. The Quality of Contraceptive Counseling (QCC) Scale is a previously validated scale that comprehensively measures individuals’ experiences receiving counseling in three subscales: Information Exchange, Interpersonal Relationship, and Disrespect and Abuse. We sought to better understand the correlation of client, provider, and visit factors with client-reported quality of contraceptive counseling in the public sector in two Mexican states using the QCC Scale.

**Methods:**

This cross-sectional survey study used the QCC Scale total score and subscale scores as outcome variables. Explanatory variables included clients’ age, LGBTTTIQ status, relationship status, number of children, education, and occupation; providers’ gender and type of provider; and the reason for visit. Linear and logistic regression models assessed bivariate associations. Multivariable, multilevel mixed-effects models with clinic as a random effect were fit. All models used complete cases (n = 470).

**Results:**

In the multilevel mixed-effects analyses, patients aged 35+ years reported worse Information Exchange (coefficient − 0.29, p = 0.01). Clients receiving care post-partum reported worse Information Exchange (coefficient − 0.25, p = 0.02) and worse total scores (coefficient − 0.15, p = 0.04) compared to clients seeking contraceptive information or methods. Clients who had 1+ children reported better Information Exchange (coefficient 0.21, p = 0.01) than those with no children. Though Disrespect and Abuse subscale scores were overall high (indicating high quality of care), we found a significant association between age and report of such negative experiences: clients in increasing age categories had increasingly higher adjusted odds of reporting no disrespect and abuse (aORs compared to the youngest group were 2.50 for those aged 19–24 years, p = 0.04; 4.53 for those 25–34 years, p = 0.01; and 6.11 for those 35+ years, p = 0.01.)

**Conclusions:**

Our findings align with previous results that younger clients have lower adjusted odds of reporting high-quality services in Mexico. There is a need for continued work supporting youth-friendly services in Mexico, and efforts should aim to ensure zero tolerance for disrespectful or coercive provider behaviors, such as pressuring or scolding clients. Improvements are also needed to ensure quality in counseling for post-partum clients, those aged 35+ years, and those without children.

## Plain English summary

Monitoring clients’ experiences with contraceptive care is vital to help improve service delivery and make sure individuals’ human rights are fulfilled. The Quality of Contraceptive Counseling (QCC) Scale is a survey which measures individuals’ experiences receiving counseling about contraceptives. It is grounded in principles of person-centeredness and human rights and asks directly about negative experiences. This paper describes an analysis of data collected using the QCC Scale, exploring statistical associations between the QCC Scale scores and the client, provider, and visit characteristics. The study also analyses scores from the three QCC subscales (Information Exchange, Interpersonal Relationship, and Disrespect and Abuse). The results of the analyses revealed that, though it was not commonly reported, younger clients had higher odds of reporting Disrespect and Abuse. Clients aged 35+ years reported worse Information Exchange, clients receiving care post-partum reported both worse Information Exchange and worse total scores, and clients who had 1+ children reported better Information Exchange. These findings align with previous results that younger clients have lower odds of reporting high-quality services in Mexico. There is a need for continued work supporting youth-friendly services in Mexico, and efforts should aim to reduce disrespectful or coercive provider behaviors, such as pressuring or scolding clients. Improvements are also needed to ensure quality in counseling for post-partum clients, those aged 35+ years, and those without children.

## Resumen

**Antecedentes:**

Monitorear las experiencias de las usuarias con los servicios anticonceptivos es vital para visibilizar los esfuerzos orientados a mejorar la calidad de la atención y asegurar que sus derechos humanos sean respetados. La Escala para Medir la Calidad de la Consejería Anticonceptiva (QCC por sus siglas en inglés) es un instrumento previamente validado que mide exhaustivamente las experiencias individuales de las usuarias al recibir una orientación consejería considerando tres subescalas: Intercambio de Información, Relaciones Interpersonales y Abuso y Maltrato. Con este artículo buscamos entender la correlación de las características de las usuarias, del personal de salud y de la consulta con la calidad de la consejería anticonceptiva reportada en la Escala QCC en el sector público en dos estados mexicanos.

**Método:**

Se trata de un estudio transversal de encuesta. Se analizaron las puntuaciones totales y de las subescalas de la Escala QCC como variables de salida. Las variables explicativas incluyeron las siguientes características de las usuarias: edad, identificación LGBTTTIQ, su estatus relacional, número de hijos, nivel educativo y ocupación; las características del personal de salud: género y formación; y por último, el motivo de consulta. Empleamos modelos de regresión linear y logística para evaluar asociaciones bivariadas. Se ajustaron modelos multivariado y multinivel de efectos mixtos, con la clínica como efecto aleatorio. Únicamente se consideraron los casos completos (n = 470) para todos los modelos.

**Resultados:**

En el análisis multinivel de efectos mixtos, las usuarias de 35 o más años reportaron peor Intercambio de Información (coeficiente − 0.29, p = 0.01). Las usuarias que recibieron atención postparto reportaron peor Intercambio de Información (coeficiente −0.25, p = 0.02) y peor puntaje total (coeficiente − 0.15, p = 0.04) en comparación con las usuarias cuyo motivo de consulta era buscar métodos anticonceptivos o información al respecto. Las usuarias que tenían uno o más hijos reportaron mejor Intercambio de Información (coeficiente 0.21, p = 0.01) en contraste con aquellas que no tenían hijos. Aunque los puntajes de las subescalas fueron altos en general (lo cual indica que la calidad de la atención es alta), encontramos una asociación significativa entre la edad y experiencias negativas: a medida que los grupos de edad de las usuarias incrementan, también incrementa la probabilidad de no reportar Abuso o Maltrato (aORs comparado con el grupo de edad más joven fue 2.50 para el grupo de 19-24 años, p = 0.04; 4.53, para el grupo de 25-34 años, p = 0.01; y 6.11 para el grupo 35+ años, p = 0.01.)

**Conclusiones:**

Nuestros hallazgos coinciden con resultados previos que indican que las usuarias más jóvenes tienen menos probabilidades de reportar valores altos de calidad de los servicios anticonceptivos en México. Es necesario continuar trabajando y apoyando a los servicios amigables para adolescentes en México, y los esfuerzos se deberían enfocar en asegurar tolerancia cero de comportamientos irrespetuosos o coercitivos por parte del personal de salud, tales como presionar o regañar a las usuarias. También es necesario mejorar y garantizar la calidad de la orientación consejería para usuarias en postparto, aquellas que tienen 35 o más años, y aquellas que no tienen hijos.

## Resumen Simple

Monitorear las experiencias de las usuarias con los servicios anticonceptivos es vital para visibilizar los esfuerzos orientados a mejorar la calidad de la atención y garantizar sus derechos humanos. La Escala para Medir la Calidad de la Consejería Anticonceptiva (QCC) es una encuesta que evalúa las experiencias individuales de las usuarias con los servicios anticonceptivos. Está fundamentada en perspectivas centrada en la persona y de derechos humanos, e indaga explícitamente sobre posibles experiencias negativas. Este artículo describe el análisis de los datos recabados de la implementación de la Escala QCC, explorando asociaciones estadísticas entre las puntuaciones de las características de las usuarias, personal de salud y visita. En este estudio, también se analizaron los puntajes de las tres subescalas que conforman la Escala QCC (Intercambio de Información, Relaciones Interpersonales y Maltrato y Abuso). Los resultados del análisis revelan que, aunque no es reportado comúnmente, las usuarias más jóvenes tienen más probabilidades de reportar Abuso y Maltrato. Las usuarias de 35 o más años reportaron peor Intercambio de Información; las usuarias que recibieron atención postparto reportaron peor Intercambio de Información y peores puntuaciones totales; y las usuarias que tenían uno o más hijos, reportaron tener mejor Intercambio de Información. Estos hallazgos coinciden con resultados previos que indican que las usuarias más jóvenes tienen menores razones de probabilidad ajustada de reportar valores altos de calidad de los servicios anticonceptivos en México. Es necesario seguir apoyando a los servicios amigables para adolescentes en México, y los esfuerzos deberían apuntar a eliminar conductas irrespetuosas o coercitivas del personal de salud, tales como presionar o regañar a las usuarias. También es necesario mejorar y garantizar la calidad de la orientación consejería para usuarias en postparto, aquellas que tienen 35 o más años, y aquellas que no tienen hijos.

## Background

High quality contraceptive services empower people to control the number of children they will have and the spacing of their pregnancies, thereby securing a basic human right [1]. This critical aspect of health care requires quality monitoring and improvement efforts grounded in human rights principles, particularly given the history of coercion, abuse, and oppressive policies that family planning carries [2–6]. Associations between subgroups of clients,^1^ providers, or visits and the quality of care received should be explored, both to shape programmatic priorities and to guard against inequities in the quality of contraceptive services provided.

A vital part of providing contraception, and thus an important area in which to evaluate quality, is contraceptive counseling. Contraceptive counseling is the discussion of contraceptive methods, including any conversation with a service provider covering initiation, continuation, cessation, or information about a method [7–9]. Counseling has been found to correlate with use and continuation of contraceptives, though the association is not always clear [10–12]. Importantly, traditional measures such as increased use and continuation do not adequately capture the goals of human rights-based contraceptive services [13, 14]. Thus, better measures which focus on client experience and the support given to make fully-informed, voluntary decisions, should be used to evaluate contraceptive counseling [15–17].

In 2017 Holt, Dehlendorf, and Langer created a framework for contraceptive counseling quality grounded in quality of care and human rights principles and research in healthcare communication [15]. The framework divides the counseling process into three stages (needs assessment, decision-making support, and method choice and follow-up), detailing technical, interpersonal, and relationship-building elements that are necessary throughout the process. Based on this framework, Holt et al. developed and validated the Quality of Contraceptive Counseling (QCC) Scale, which covers three interrelated aspects of counseling quality: Information Exchange, Interpersonal Relationship, and Disrespect and Abuse [15, 18]. The scale comprehensively measures aspects of the counseling process related not only to information receipt but also individuals’ experiences having the opportunity to participate in the method selection process and the degree to which they had positive and trusting experiences with the provider. The Disrespect and Abuse subscale, and its inclusion of direct questions about negative experiences, makes this scale unique [19], and provides a tool to support human rights in reproductive care, which is of particular importance given the history of coercion and abuse in the field. The scale is designed for clients to evaluate care, and thus emphasizes client experience.

Developments in health care provision in Mexico over recent decades provide an excellent setting for an exploration of factors relating to quality in contraceptive counseling. Mexico’s history of health reform includes the creation of the Ministry of Health and the Mexican Institute for Social Security (Instituto Mexicano del Seguro Social) in 1943, reforms in the 1970s focusing on adopting a primary health-care paradigm and extending coverage through decentralization, and the 2003 legislation creating a System of Social Protection in Health (Sistema de Protección Social en Salud), a system which frames healthcare as a right, both legally and ethically, for all Mexican citizens [20, 21]. This includes the creation of a Popular Health Insurance scheme (Seguro Popular, SP) to extend health care coverage for those not covered under social security, including the unemployed, self-employed, and agricultural workers [22]. The reforms have been implemented through monitoring and research. A commitment to a “rigorous programme of evidence generation, monitoring and assessment” is continually affirmed by policy makers [20, 21, 23]. Quality is emphasized in one of the main tenants of the reform (“protection of patients through quality assurance of health care”) and is operationalized through an accreditation process and the monitoring of several quality indicators [21, 24]. Expansion in reproductive health services have figured prominently in this reform, so together the emphasis on quality, evidence, and family planning services creates an ideal backdrop for this study [22, 25].

While related research into contraceptive services quality correlates from Mexico does exist, previous studies have used single item measures or unvalidated composites of survey questions to assess quality. Our study, investigating factors related to quality as measured by a validated and detailed measurement of quality, is unique. It builds on Darney et al.’s 2016 finding from a nationally representative survey in Mexico that women 15–19 and 20–24 years old had lower odds of reporting high-quality contraceptive services than women 25–29 years (high-quality being operationalized as a “yes” response to five quality items from a larger survey) [26]. Other factors associated with reporting lower quality in this study were living in an Indigenous household or having fewer years of education that expected for one’s age, while covariates associated with reporting high quality were receiving the requested method of contraception and being in the highest wealth quintile. Slater et al.’s [27] cross-sectional survey of 18 public clinics found a different relationship between age and satisfaction, noting that clients ages 20–35 years reported lower levels of satisfaction than adolescents and clients over 35 years, though it is important to note that this study focused on a single question outcome of overall satisfaction rather than a composite description of quality. Other factors associated with reporting satisfaction in this study, most of which were in fact aspects of quality, were receiving sufficient information, feeling that opinions were taken into consideration, feeling that motives were addressed, having enough time in the consultation, being able to ask questions, experiencing few interruptions, feeling satisfied with the provided method, and having previously been pregnant or having had a partner become pregnant.

Associations between quality and characteristics of clients, such as age, education, indigeneity, and reproductive status, could represent significant health disparities and indicate the need for further research. This paper seeks to investigate correlations between client-reported quality of contraceptive counseling and client, provider, and visit factors in two Mexican states utilizing the validated, client-centered, and human rights-based QCC Scale.

## Methods

This analysis uses secondary data from an observational, cross-sectional survey conducted with the primary purpose of developing and validating the QCC Scale [18]. The exploratory analysis examined factors associated with client-reported quality of contraceptive counseling as measured by the QCC Scale. Factors included characteristics of the clients, providers, and visits. Quality was measured by the QCC Scale’s total composite score and each of its three subscale scores: Information Exchange, Interpersonal Relationship, and Disrespect and Abuse.

The Harvard T. H. Chan School of Public Health Institutional Review Board gave approval for the original study (IRB16-1176), and a local advisory board in Mexico comprised of three individuals not involved in the research also reviewed and approved the study. This secondary study was conducted using a de-identified dataset and thus did not require ethics approval, as confirmed through the UC Berkeley Committee for Protection of Human Subjects.

In order to ensure a wide range of participants, clients were recruited from a convenience sample of clinics in two states in Mexico: the urban and mostly progressive capital of Mexico City, and the more conservative state of San Luis Potosí (which includes both urban and rural municipalities). The sample included eight public clinics in each state and two public hospitals in San Luis Potosí, all under the jurisdiction of the Health Secretariat in Mexico, for a total of 18 sites. The Health Secretariat in Mexico provides services to the population not employed in the formal sector and policy ensures a full range of contraceptive methods should be available without cost.

### Sample

**Fig. 1:**
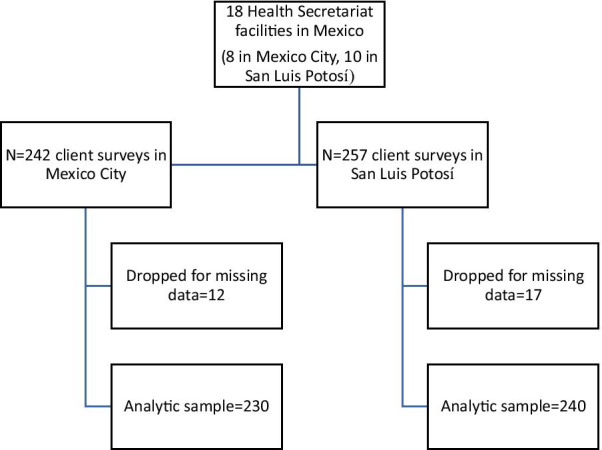
Sample flow chart

Eligibility criteria were being women (as assessed by interviewers) and having spoken with a provider about contraception on the day of recruitment. We conducted 242 exit interviews in Mexico City and 257 in San Luis Potosí (N = 499) and 95% of participants were recruited in public clinics (versus 5% in hospitals). The sample size target was based on the primary outcome of the study and requirements for exploratory factor analysis utilized to construct the QCC Scale [18]. From the total sample size of 499, 29 cases (about 5.8%) were dropped due to missing data, leaving a sample size of n = 470 for the analyses (Fig. 1).

### Data collection

Recruitment took place between September 2016 and July 2017. Interviewers, who were members of the Mexfam research and evaluation team and were trained by the investigators, approached all clients who appeared to be women of reproductive age to invite them to participate in an exit interview. Additionally, receptionists gave out flyers and directed clients to interviewers. After receiving informed consent from participants, the original 35-item QCC Scale item pool [18] was administered verbally in private areas of the study sites. Participants’ background characteristics and reasons for visits were also recorded.

### Measures

Outcome variables for this analysis included the three subscale scores from the final QCC Scale (two continuous and one dichotomous) and the total score (continuous). The Information Exchange subscale (continuous) consists of ten items, the Interpersonal Relationship subscale (continuous) consists of seven items, and the Disrespect and Abuse subscale consists of five items (recoded to be dichotomous using top-scoring because the distribution was too strongly skewed [18]) (Table 1). Item responses were given on four-point Likert scales. Response categories for positively worded items were “completely agree/totalmente de acuerdo” (4), “agree/de acuerdo” (3), “disagree/en desacuerdo” (2), and “completely disagree/totalmente en desacuerdo” (1). Response categories for negatively worded items were “yes/sí” (1), “yes with doubts/sí con dudas” (2), “no with doubts/no con dudas” (3), and “no/no” (4). Composite subscale and total scores were calculated as mean scores.

**Table 1:** Item description, Quality of Contraceptive Counseling (QCC) Scale and Subscales (N = 499, QCC Mexico Survey)

Original Spanish wording followed by English translation	Mean (SD)
Information Exchange subscale	3.3 (0.6)
1. Durante la consulta sobre métodos anticonceptivos, pude opinar sobre mis necesidades	3.5 (0.6)
*During the contraception consultation, I was able to give my opinion about what I needed*	
2. Recibí información completa sobre mis opciones para el uso de métodos anticonceptivos	3.5 (0.7)
*I received complete information about my options for contraceptive methods*	
3. El/la prestadora de servicios de salud supo explicar claramente los métodos anticonceptivos	3.4 (0.7)
*The provider knew how to explain contraception clearly*	
4. Tuve la oportunidad de participar en la elección de un método anticonceptivo	3.6 (0.6)
*I had the opportunity to participate in the selection of a method*	
5. Recibí información sobre cómo protegerme de una infección de transmisión sexual	3.3 (0.9)
* I received information about how to protect myself from sexually transmitted infections*	
6. Me dijeron qué hacer si falla un método anticonceptivo (e.j., condón roto, olvido de pastilla, sentir el DIU mal colocado)	2.9 (0.9)
* I received information about what to do if a method fails (e.g., broken condom, forget a pill, feel an IUD is poorly placed)*	
7. Pude entender las reacciones que podría tener mi cuerpo al usar un método anticonceptivo	3.3 (0.8)
* I could understand how my body might react to using contraception*	
8. Pude entender cómo usar el método o los métodos anticonceptivos de los que hablamos	3.4 (0.7)
* I could understand how to use the method(s) we talked about during the consultation*	
9. Recibí información sobre qué hacer si quisiera dejar de usar un método anticonceptivo	3.2 (0.8)
* I received information about what to do if I wanted to stop using a method*	
10. Me explicaron qué hacer si tenía una reacción al método anticonceptivo (e.j., alergia, nauseas, cólicos, alteraciones en la menstruación)	3.1 (0.9)
* The provider explained to me what to do if I had a reaction to a method (e.g., allergies, nausea, pains, menstrual changes)*	
Interpersonal Relationship subscale	3.6 (0.5)
11. Sentí que la información que proporcioné iba a quedar entre el/la prestadora de servicios de salud y yo	3.6 (0.6)
* I felt the information I shared with the provider was going to stay between us*	
12. Sentí que el/la prestadora de servicios de salud me daba el tiempo necesario para explorar mis opciones sobre métodos anticonceptivos	3.5 (0.6)
* The provider gave me the time I needed to consider the contraceptive options we discussed*	
13. El/la prestadora de servicios de salud me brindó un trato amable durante la consulta sobre métodos anticonceptivos	3.7 (0.6)
* The provider was friendly during the contraception consultation*	
14. Sentí que el/la prestadora de servicios de salud tenía conocimiento sobre los métodos anticonceptivos	3.7 (0.5)
* I felt the health care provider had sufficient knowledge about contraceptive methods*	
15. El/la prestadora de servicios de salud se interesó por mi salud al platicar sobre métodos anticonceptivos	3.5 (0.6)
* The provider showed interest in my health while we talked about contraception*	
16. El/la prestadora de servicios de salud se interesó por lo que yo pine	3.6 (0.6)
* The provider was interested in my opinions*	
17. Me sentí escuchada por el/la prestadora de servicios de salud	3.6 (0.6)
* I felt listened to by the provider*	
Disrespect and Abuse subscale	3.9 (0.4)
18. El/la prestadora de servicios de salud me insistió para usar el método anticonceptivo que él/ella quería	3.9 (0.6)
* The provider pressured me to use the method they wanted me to use*	
19. Sentí que el/la prestadora de servicios de salud me atendió mal debido a que suele juzgar a las personas	3.9 (0.4)
* I felt the provider treated me poorly because they tend to judge people*	
20. Sentí que me regañaban por mi edad	3.9 (0.6)
*I felt scolded because of my age*	
21. El/la prestadora de servicios de salud me hizo sentir incómoda por mi vida sexual (e.j., inicio de vida sexual, preferencia sexual, número de parejas, número de hijos)	3.9 (0.6)
*The provider made me feel uncomfortable because of my sex life (e.g., when I started having sex, my sexual preferences, the number of partners I have, the number of children I have)*	
22. El/la prestadora de servicios de salud me observó o me tocó de una forma que me hizo sentir incómoda	4.0 (0.3)
*The provider looked at me or touched me in a way that made me feel uncomfortable*	
Overall composite score	3.5 (0.4)

Item Description, QCC Scale and Subscales. Higher scores represent higher quality. Response categories for positively worded items were “completely agree/totalmente de acuerdo” (4), “agree/de acuerdo” (3), “disagree/en desacuerdo” (2), and “completely disagree/totalmente en desacuerdo” (1). Response categories for negatively worded items were “yes/sí” (1), “yes with doubts/sí con dudas” (2), “no with doubts/no con dudas” (3), and “no/no” (4). Missing data ranges from 0–6 cases per item, except for item 4 (missing 47 cases), which had a “not applicable” option. Some of these data were previously published in Holt et al.’s “Development and Validation of the Client-Reported Quality of Contraceptive Counseling Scale” [18]

*SD* standard deviation

Given the exploratory nature of this study, all available characteristics of the clients, providers, and visits which were hypothesized to have a possible association with reported quality of care were included as explanatory categorical variables in the analyses. These included clients’ age, LGBTTTIQ (lesbian, gay, bisexual, transgendered, transexual, two-spirited, intersexed, queer) status, relationship status, number of children, education, occupation; providers’ gender and type of provider; and the reason for visit. Participants were coded as LGBTTTIQ if at least one of the following was true: for sexual orientation they chose women or both (options were men, women, or both); or they self-identified by responding yes to the question, “Do you identify as part of the sexually diverse community (LGBTTTIQ)?” (original Spanish question, “¿Se identifica como parte de la comunidad de la diversidad sexual (LGBTTTIQ)?”). Clients’ characteristics were hypothesized to affect the reported quality of counseling through potential discrimination on the part of the provider or due to differences in clients’ expectations. Similarly, providers’ characteristics could potentially affect provider biases and care provided. Visits’ characteristics could affect care given based on different kinds of visits having differing norms or protocols, or there could be provider biases towards clients coming in for different kinds of visits.

For use in an additional exploratory analysis that was added to the study, each of the five questions on the Disrespect and Abuse subscale (items 18–22 in Table 1) were dichotomized as either top-scoring (response of 4) or other (response of 0–3).

### Statistical analyses

We assessed bivariate associations between our explanatory variables and each of the four quality scores using linear regressions for the three continuous outcomes and logistic regressions for the dichotomous outcome.

We built multivariable models using our nine explanatory variables of interest with each of the outcomes, again creating three models with linear regression for the continuous outcomes and one with logistic regression for the dichotomous outcome. To account for clustering of clients within clinics, each of the multivariable models was a multilevel mixed-effects model with clinic as a random effect. The intraclass correlation (ICC) for clinic was calculated for each of the four multivariable models to assess the amount of the total variance in quality scores that was due to differences at the clinic level. We attempted to include provider as a random effect, but due to a combination of missingness in this variable and having small numbers of clients for some of the providers, maximum likelihood estimation could not converge, thus we could not account for nesting of clients within specific providers. Null multilevel models were also created for each outcome (using clinic as a random effect but no explanatory variables in the model).

For any categorical variable with more than two levels, we ran an overall Wald test to compare all levels (rather than just comparing each with the reference level), calculating a Chi-squared test statistic and corresponding p-value. In this test, a p-value less than 0.05 confirms that there are significant differences among some levels of the variable.

To inform recommendations for potential quality improvement interventions, based on initial results, an additional exploratory analysis of the individual items on the Disrespect and Abuse subscale was added to the study. Each item was used as one variable along with age category as the other variable in a two-way table. Because we anticipated small expected cell counts, Fisher’s exact tests were used to assess the association between the items and the age categories.

All tests used complete case analyses and alpha = 0.05 as a cutoff for statistical significance. All analyses were conducted using Stata SE 15.1 [28]. The Stata commands that were used for the bivariate models were “logit” for the binary and “regress” for the continuous outcomes. For the multivariable multilevel models, “melogit” was used for binary and “mixed” for continuous outcomes, both followed with the “estat” command for ICC calculations. Finally, for the additional analyses of individual items on the Disrespect and Abuse subscale, “tab…, row exact” was used.

## Results

### Participant, provider, and visit characteristics

Participants were diverse by age category and relationship status (Table 2). They were mostly non-LGBTTTIQ identifying (91%), had at least one child (75%), and their occupation was dedicated to housework or other unpaid work (63%). Providers were mostly female (73%) and doctors (71%). Visit types varied, with about one-third (37%) seeing the provider to request a contraceptive or to seek contraceptive information. Other reasons for the visits included consulting about their current method (19%), requesting removal of their current method (8%), prenatal consults (14%), post-partum visits (9%), and “other” care (13%) including preventative checkups, post-abortion care, and specialty care.

**Table 2:** Provider, participant, and visit characteristics (QCC Mexico Survey)

Characteristic	n	%
Total	470	100.0
Age (min: 15, max: 51, avg: 26.2)		
15–18 years	72	15.3
19–24 years	176	37.5
25–34 years	137	29.2
35+ years	85	18.1
LGBTTTIQ status		
Not LGBTTTIQ	427	90.9
LGBTTTIQ^a^	43	9.1
Relationship status		
Single	120	25.5
In a relationship	212	45.1
Married	119	25.3
Divorced, widowed, or separated	19	4.0
Children (min: 0, max: 5 avg: 1.3)		
None	119	25.3
1+	351	74.7
Education		
Primary school or less	57	12.1
Secondary school	217	46.2
More than secondary school	196	41.7
Occupation		
Work for pay	109	23.2
Work without pay	298	63.4
Student	63	13.4
Provider gender		
Female	345	73.4
Male	125	26.6
Provider type		
Doctor	335	71.3
Nurse	123	26.2
Social worker or other^b^	12	2.6
Reason for visit		
Request contraceptive or ask for information	172	36.6
Prenatal consult	66	14.0
Remove method	39	8.3
Method follow-up	90	19.2
Post-partum	42	8.9
Other^c^	61	13.0

^a^Responded that they identified as part of the LCBTTTIQ (lesbian, gay, bisexual, transgendered, transsexual, two-spirited, intersexed, queer) community; or responded that they were attracted to women or both men and women

^b^Other provider types included social workers, psychologists, and health promoters

^c^Other reasons for visit included preventative checkups, post-abortion care, and other specialty care

### Bivariate analyses

In bivariate analyses (Table 3), participants who had children reported higher Information Exchange scores (coefficient 0.15, p = 0.02), higher Interpersonal Relationship scores (coefficient 0.10, p = 0.05), and higher total scores (coefficient 0.10 p = 0.02) compared to those with no children. Participants whose reason for visit fit under the “other” category (i.e., preventative checkups, post-abortion care, and specialty care) reported lower Information Exchange scores (coefficient − 0.16, p = 0.05) and lower Interpersonal Relationship scores (coefficient − 0.014, p = 0.04) compared to those whose primary reason for the visit was to seek a contraceptive method or information about contraceptives. Participants who worked without pay reported higher Interpersonal Relationship scores (coefficient 0.12, p = 0.03) compared to those who worked for pay. Compared to those in the youngest age category, participants in every increasing age category had higher odds of reporting no disrespect and abuse (odds ratios (aORs) for increasing age categories were 2.22, 4.23, and 5.33, respectively; all with p-values < 0.03). The coefficients reported for differences in Information Exchange, Interpersonal Relationship, and total scores represent a difference in the mean scores between the two groups, where mean scores could theoretically range from 1.00 to 4.00 (see Table 1 for overall mean scores).

**Table 3:** Unadjusted bivariate regressions results for subscale scores and total scores and sample characteristics (QCC Mexico Survey)

Variables	Information Exchange Score^a^	Interpersonal Relationship Score^a^	Disrespect and Abuse Score^a^	Total Score^a^
	Coefficient (95% CI)	Coefficient (95% CI)	Odds ratio (95% CI)	Coefficient (95% CI)
Age (years)				
15–18	REF	REF	REF	REF
19–24	− 0.072 (− 0.226 to 0.081)	− 0.009 (− 0.140 to 0.121)	2.217 (1.112 to 4.423)*	− 0.018 (− 0.128 to 0.092)
25–34	− 0.015 (− 0.175 to 0.145)	− 0.011 (− 0.124 to 0.147)	4.233 (1.835 to 9.767)**	0.025 (− 0.090 to 0.139)
35+	− 0.138 (− 0.314 to 0.038)	− 0.097 (− 0.246 to 0.052)	5.333 (1.868 to 15.229)**	− 0.071 (− 0.197 to 0.055)
LGBTTTIQ status				
Not LGBTTTIQ	REF	REF	REF	REF
LGBTTTIQ	0.060 (− 0.116 to 0.236)	0.076 (− 0.072 to 0.225)	2.968 (0.698 to 12.623)	0.057 (− 0.0687 to 0.183)
Relationship status				
Single	REF	REF	REF	REF
In a relationship	0.032 (− 0.092 to 0.158)	0.081 (− 0.024 to 0.187)	1.054 (0.543 to 2.046)	0.038 (− 0.051 to 0.128)
Married	0.127 (− 0.015 to 0.269)	0.070 (− 0.050 to 0.190)	1.880 (0.796 to 4.441)	0.081 (− 0.021 to 0.182)
Divorced, widowed, or separated	− 0.137 (− 0.408 to 0.134)	− 0.158 (− 0.387 to 0.071)	0.577 (0.170 to 1.958)	− 0.139 (− 0.333 to 0.054)
Children				
None	REF	REF	REF	REF
1+	0.145 (0.029 to 0.261)*	0.099 (0.000 to 0.197)*	1.468 (0.802 to 2.685)	0.099 (0.016 to 0.182)*
Education				
Primary school or less	REF	REF	REF	REF
Secondary school	0.077 (− 0.086 to 0.241)	− 0.001 (− 0.137 to 0.139)	0.421 (0.143 to 1.241)	− 0.027 (− 0.090 to 0.144)
More than secondary school	− 0.026 (− 0.140 to 0.191)	− 0.077 (− 0.217 to 0.063)	0.703 (0.229 to 2.157)	− 0.021 (− 0.139 to 0.098)
Occupation				
Work for pay	REF	REF	REF	REF
Work without pay	0.091 (− 0.032 to 0.214)	0.115 (0.012 to 0.219)*	0.961 (0.478 to 1.931)	0.077 (− 0.011 to 0.165)
Student	0.076 (− 0.098 to 0.250)	0.067 (− 0.080 to 0.214)	0.656 (0.266 to 1.618)	0.041 (− 0.083 to 0.165)
Provider gender				
Female	REF	REF	REF	REF
Male	− 0.044 (− 0.159 to 0.071)	0.027 (− 0.070 to 0.125)	0.736 (0.403 to 1.344)	− 0.008 (− 0.090 to 0.074)
Provider type				
Doctor	REF	REF	REF	REF
Nurse	0.031 (− 0.086 to 0.147)	− 0.025 (− 0.123 to 0.074)	1.540 (0.768 to 3.087)	0.019 (− 0.064 to 0.102)
Social worker or other	0.001 (− 0.323 to 0.324)	− 0.115 (− 0.388 to 0.159)	1.663 (0.210 to 13.201)	− 0.030 (− 0.262 to 0.201)
Reason for visit				
Request contraceptive or ask for information	REF	REF	REF	REF
Prenatal consult	− 0.133 (− 0.290 to 0.024)	− 0.044 (− 0.178 to 0.090)	0.592 (0.271 to 1.292)	− 0.076 (− 0.189 to 0.037)
Remove method	0.079 (− 0.113 to 0.271)	− 0.018 (− 0.146 to 0.182)	1.579 (0.271 to 1.292)	0.047 (− 0.091 to 0.185)
Method follow-up	0.136 (− 0.005 to 0.277)	0.060 (− 0.061 to 0.180)	1.349 (0.569 to 3.196)	0.083 (− 0.018 to 0.185)
Post-partum	− 0.177 (− 0.364 to 0.010)	− 0.099 (− 0.258 to 0.061)	0.658 (0.258 to 1.677)	− 0.121 (− 0.254 to 0.013)
Other^b^	− **0.164 (**− **0.325 to **− 0.002)*	− **0.142 (**− **0.279 to **− 0.004)*	1.206 (0.460 to 3.160)	− 0.113 (− 0.229 to 0.003)

^*^p-value < 0.05, **p-value < 0.01

^a^Higher score represents higher quality (including Disrespect and Abuse subscale, where higher score indicates less disrespect)

^b^Other reasons for visit included preventative checkups, post-abortion care, and other specialty care

### Multivariable multilevel mixed-effects analyses

The results of all multivariable analyses are shown in Table 4. In the mixed effects linear regression model with Information Exchange Score as an outcome, two associations were statistically significant at the alpha = 0.05 level. Participants receiving care post-partum reported worse Information Exchange scores (coefficient − 0.25, p = 0.02) compared to those whose reason for visit was to request a contraceptive method or seek more information about a method, controlling for all other variables in the model. Also, participants in the oldest age category, 35+ years, reported worse Information Exchange scores (coefficient − 0.29, p = 0.01) compared to those in the youngest category, 15–18 years, while controlling for all other variables in the model, although an overall Wald test to detect differences at any level of the age category variable revealed that this finding is borderline, with Wald test p-value of 0.057.

**Table 4:** Multivariable multilevel regressions results for subscale and total scores and sample characteristics (QCC Mexico Survey)

Variables	Information Exchange Score^a^	Interpersonal Relationship Score^a^	Disrespect and Abuse Score^a^	Total score^a^
	Coefficient (95% CI)	Coefficient (95% CI)	Adjusted odds ratio (95% CI)	Coefficient (95% CI)
Age (years)				
15–18	REF	REF	REF	REF
19–24	− 0.153 (− 0.322 to 0.016)	− 0.045 (− 0.189 to 0.099)	2.495 (1.06 to 5.873)*	− 0.058 (− 0.178 to 0.063)
25–34	− 0.146 (− 0.331 to 0.038)	− 0.037 (− 0.194 to 0.120)	4.526 (1.577 to 12.994)**	− 0.041 (− 0.173 to 0.091)
35+	− 0.287 (− 0.496 to − 0.078)**	− 0.145 (− 0.323 to 0.033)	6.105 (1.647 to 22.631)**	− 0.147 (− 0.296 to 0.002)
LGBTTTIQ status				
Not LGBTTTIQ	REF	REF	REF	REF
LGBTTTIQ	0.074 (− 0.102 to 0.250)	0.082 (− 0.069 to 0.233)	3.68 (0.792 to 17.095)	0.068 (− 0.058 to 0.195)
Relationship status				
Single	REF	REF	REF	REF
In a relationship	− 0.019 (− 0.166 to 0.127)	0.015 (− 0.110 to 0.139)	0.836 (0.360 to 1.945)	− 0.017 (− 0.122 to 0.088)
Married	0.066 (− 0.107 to 0.239)	0.007 (− 0.141 to 0.154)	0.899 (0.296 to 2.731)	0.012 (− 0.112 to 0.136)
Divorced, widowed, or separated	− 0.176 (− 0.457 to 0.104)	− 0.167 (− 0.406 to 0.071)	0.236 (0.052 to 1.065)	− 0.176 (− 0.376 to 0.024)
Children				
None	REF	REF	REF	REF
1+	0.210 (0.059 to 0.361)**	0.122 (− 0.006 to 0.251)	0.994 (0.405 to 2.439)	0.132 (0.024 to 0.24)*
Education				
Primary school or less	REF	REF	REF	REF
Secondary school	0.079 (− 0.080 to 0.238)	0.006 (− 0.129 to 0.141)	0.443 (0.139 to 1.409)	0.032 (− 0.081 to 0.145)
More than secondary school	0.066 (− 0.099 to 0.232)	− 0.062 (− 0.203 to 0.079)	0.687 (0.201 to 2.347)	0.004 (− 0.114 to 0.122)
Occupation				
Work for pay	REF	REF	REF	REF
Work without pay	0.042 (− 0.092 to 0.177)	0.072 (− 0.042 to 0.187)	1.272 (0.537 to 3.012)	0.046 (− 0.050 to 0.142)
Student	0.046 (− 0.141 to 0.234)	0.076 (− 0.084 to 0.235)	0.898 (0.294 to 2.742)	0.033 (− 0.101 to 0.167)
Provider gender				
Female	REF	REF	REF	REF
Male	− 0.039 (− 0.161 to 0.083)	0.022 (− 0.083 to 0.127)	0.863 (0.421 to 1.770)	0.00 (− 0.088 to 0.088)
Provider type				
Doctor	REF	REF	REF	REF
Nurse	0.014 (− 0.128 to 0.156)	0.000 (− 0.126 to 0.126)	1.682 (0.690 to 4.099)	0.032 (− 0.075 to 0.139)
Social worker or other	− 0.051 (− 0.382 to 0.280)	− 0.051 (− 0.382 to 0.280)	3.420 (0.347 to 33.737)	− 0.063 (− 0.301 to 0.175)
Reason for visit				
Request contraceptive or ask for information	REF	REF	REF	REF
Prenatal consult	− 0.118 (− 0.280 to 0.044)	− 0.042 (− 0.180 to 0.097)	0.596 (0.235 to 1.510)	− 0.069 (− 0.185 to 0.048)
Remove method	0.081 (− 0.109 to 0.271)	0.03 (− 0.132 to 0.192)	1.476 (0.379 to 5.747)	0.053 (− 0.083 to 0.189)
Method follow-up	0.113 (− 0.033 to 0.259)	0.05 (− 0.075 to 0.174)	1.185 (0.455 to 3.088)	0.066 (− 0.039 to 0.171)
Post-partum	− 0.246 (− 0.444 to − 0.048)*	− 0.125 (− 0.297 to 0.046)	0.683 (0.227 to 2.061)	− 0.149 (− 0.294 to − 0.005)*
Other^b^	− 0.125 (− 0.297 to 0.047)	− 0.112 (− 0.260 to 0.036)	1.124 (0.362 to 3.496)	− 0.093 (− 0.217 to 0.031)
Intraclass correlation (ICC)				
Clinic as random effect ICC	0.029 (0.004 to 0.183)	0.049 (0.012 to 0.180)	0.056 (0.006 to 0.384)	0.056 (0.015 to 0.191)
Null model ICC	0.024 (0.002 to 0.200)	0.043 (0.010 to 0.170)	0.078 (0.0167 to 0.299)	0.049 (0.012 to 0.180)
Reduction in ICC from null	− 0.005	− 0.006	0.02	− 0.007

Higher score represents higher quality (including Disrespect and Abuse subscale, where higher score indicates less disrespect). Sample size for each model was 470 complete cases

^b^Other reasons for visit included preventative checkups, post-abortion care, and other specialty care

*p-value < 0.05, **p-value < 0.01

In the mixed effects linear regression model with Interpersonal Relationship as the outcome, none of the examined variables were found to show a significant association with the outcome.

In the mixed effects logistic regression model with the dichotomized Disrespect and Abuse score as the outcome, significant differences were seen across age categories. While controlling for all other variables in the model, compared to the youngest age category, those in each increasing age category had increasing adjusted odds of reporting no disrespect and abuse (aORs compared to the youngest group, 15–18 years old, were 2.50 for those 19–24 years old, p = 0.04; 4.53 for those 25–34 years old, p = 0.01; and 6.11 for those 35+ years old p = 0.01, Table 4). Of the five items on the Disrespect and Abuse subscale, item 18, “The provider pressured me to use the method they wanted me to use,” and item 20, “I felt scolded because of my age,” were significantly associated with age category. For both items, increasing age categories tended to have an increasing percentage of participants who answered “no” to these questions (Table 5).

**Table 5:** Bivariate results of Disrespect and Abuse items: Response to individual subscale items by age category (QCC Mexico Survey)

Age (years)	“The provider pressured me to use the method they wanted me to use.”^a^			“I felt the provider treated me poorly because they tend to judge people.”			“I felt scolded because of my age.”		
	Yes/maybe^b^	No^c^	Total	Yes/maybe	No	Total	Yes/maybe	No	Total
15–18	9	63	72	4	68	72	10	62	72
	12.5%	87.5%	100.0%	5.6%	94.4%	100.0%	13.9%	86.1%	100.0%
19–24	11	165	176	8	168	176	10	166	176
	6.3%	93.8%	100.0%	4.6%	95.5%	100.0%	5.7%	94.3%	100.0%
25–34	4	133	137	3	134	137	2	135	137
	2.9%	97.1%	100.0%	2.2%	97.8%	100.0%	1.5%	98.5%	100.0%
35+	1	84	85	3	82	85	3	82	85
	1.2%	98.8%	100.0%	3.5%	96.5%	100.0%	3.5%	96.5%	100.0%
Total	25	445	470	18	452	470	25	445	470
	5.3%	94.7%	100.0%	3.8%	96.2%	100.0%	5.3%	94.7%	100.0%
Fisher’s exact test^d^			0.009**			0.592			0.003**

Age (years)	“The provider made me feel uncomfortable because of my sex life (e.g., when I started having sex, my sexual preferences, the number of partners I have, the number of children I have).”			“The provider looked at me or touched me in away that made me feel uncomfortable.”		
	Yes/maybe	No	Total	Yes/maybe	No	Total
15–18	7	65	72	1	71	72
	9.7%	90.3%	100.0%	1.4%	98.6%	100.0%
19–24	9	167	176	3	173	176
	5.1%	94.9%	100.0%	1.7%	98.3%	100.0%
25–34	3	134	137	3	134	137
	2.2%	97.8%	100.0%	2.2%	97.8%	100.0%
35+	4	81	85	1	84	85
	4.7%	95.3%	100.0%	1.2%	98.8%	100.0%
Total	23	447	470	8	462	470
	4.9%	95.1%	100.0%	1.7%	98.3%	100.0%
Fisher’s exact test			0.127			1.000

^a^This table includes Items numbered 18–22 from the Disrespect and Abuse Subscale^b^ Responses were top-scored due to high skew. “yes**/**maybe” category includes responses 0–3: “yes/sí” (1), “yes with doubts/sí con dudas” (2), “no with doubts/no con dudas” (3)

^c^“no” category includes response 4: “no/no”

^d^Fisher’s exact test for significance of association.**p-value < 0.001

In the mixed effects linear regression model with total score as the outcome, participants who had children reported higher total scores compared to those without children (coefficient 0.132, p = 0.02) and participants receiving care post-partum reported worse total scores compared to those seeking a method or more information (coefficient − 0.15, p = 0.04).

Examination of ICC (Intraclass correlation) values revealed that differences between clinics contributed to about 3% of the variation in reported quality of Information Exchange (95% CI < 1–18%), 5% of the variation in reported quality of Interpersonal Relationship (95% CI 1–18%), 6% of the variation in reported Disrespect and Abuse (95% CI 1–38%) and 6% of the variation in reported total quality score (95% CI 1–19%) when controlling for all the other variables in the models. The multilevel models fit without covariates (null models) showed nearly the same ICC values (Table 4). This demonstrates that controlling for covariates did not change our understanding of the amount of variance attributable to differences at clinic level.

## Discussion

This analysis of correlates of QCC Scale and subscale scores identified several dimensions of quality in contraceptive counseling where clients experience differential treatment related to individual factors, including age and parity, as well as what the principal reason was for their visit. The separate dimensions of the QCC Scale scores add depth to these findings, suggesting which aspects of quality may vary.

Perhaps the most significant finding in this study is the trend between increasing age groups and a lower likelihood of reporting Disrespect and Abuse in our multivariable analysis. While the Disrespect and Abuse subscale revealed high quality overall, the fact that the youngest age group was most likely to report experiencing disrespect and abuse is significant from a human rights perspective which strives to ensure access to respectful reproductive care for all ages. The increasingly larger difference when compared to subsequently older age groups reveals a clear trend: in this study, the younger a client’s age group, the more likely they are to report experiencing disrespect and abuse in a contraceptive visit. This finding aligns with Darney et al.’s previous result that women 15–19 and 20–24 years old had lower odds of reporting high-quality contraceptive services than women 25–29 years in Mexico [26]. Darney et al.’s nationally representative study implies lower quality care experienced by younger people may be generalizable throughout Mexico, while our finding suggests an added layer of specificity: perhaps younger people are reporting lower quality in part because they are more commonly experiencing disrespect and abuse in their visits. Furthermore, the additional analysis comparing individual items on the Disrespect and Abuse subscale with age categories suggests that younger clients more often feel pressured by providers to use a particular method, and more often feel scolded due to their age. These items may reveal provider bias and underline specific behaviors that interventions should seek to change.

The other age-group finding in this study, that the oldest age group (35+ years) reported worse Information Exchange subscores as compared to the youngest age group [15–18], is also noteworthy. While younger people may be experiencing more disrespect and abuse, people from older age groups seeking contraceptive counseling may not be receiving the information they are looking for from these visits, which could represent a difference in quality and relevance of information provided, or a difference in expectations between age groups. The combination of age-related factors may help clarify Slater et al.’s survey of public clinics in Mexico which found that clients ages 20–35 years reported lower levels of satisfaction than adolescents and clients over 35 years [27]. Perhaps this middle group experiences an overlap of the differing concerns from both younger groups and older groups. Overall, our age-group findings from the QCC Scale reveal that nuanced, multifaceted measures of quality are needed to tease apart actionable conclusions about contraceptive counseling. Conclusions from this study agree with past studies that have called for interventions to support youth-friendly services in Mexico, show a need for continued work in this area [29–31], and suggest that interventions need to support client-centered counseling and efforts to reduce scolding in counseling. Additional studies should be conducted to understand what sort of information clients in the 35+ years age group are seeking from their contraceptive counseling visits.

The finding in this study that people who have children reported higher Information Exchange subscores and higher total scores aligns with the Slater et al.’s finding that people who have previously been pregnant or have had a partner who was pregnant report higher satisfaction with family planning services [27]. Several explanations for this finding are plausible. Perhaps people who have previous experience with pregnancy and children have clearer perspectives and expectations about contraceptive counseling or more experience to better navigate their visit. On the other hand, perhaps providers have biases about discussing contraception with clients who do not have children.

The experiences of people receiving contraceptive counseling in post-partum visits are important to highlight. Compared to those visiting a provider for the purpose of obtaining a contraceptive method or more information about contraceptives, people receiving counseling about contraceptives in post-partum visits reported worse Information Exchange subscores and worse total quality scores in this study. This highlights an area for significant improvement in contraceptive counseling. The right to make a fully-informed, voluntary decision about contraception certainly applies to clients who have recently given birth, but some studies suggest clients are being pressured to accept LARCs immediately postpartum [32, 33]. The lower Information Exchange subscores in our study could reflect a similar focus on LARCs rather than the provision of comprehensive contraceptive counseling postpartum. Future studies should investigate clients’ qualitative experiences of contraceptive counseling postpartum in Mexico, and interventions should explore dedicating time, resources, and training to providing well-rounded contraceptive counseling and options to post-partum clients.

Multilevel analysis indicated that differences between clinics can account for around 3–6% of the variation in quality reported on each of the scales, though confidence intervals were wide. This supports the claim that differences between clinics do not adequately explain differences in reported quality, and it supports efforts to identify factors that may better explain disparities.

While the differences reported above are statistically significant, the sizes of the coefficients are small. Still, given the exploratory nature of this study, the likelihood that courtesy bias may be causing an underestimation of effects, and the fact that differences in scores between groups may represent bias and differential treatment of clients while they attempt to access critical reproductive healthcare, we argue that these findings are all notable and worthy of further discussion.

Due to the convenience sample of clients, a limitation of this study is that it is not a nationally representative sample of contraceptive counseling clients in Mexico, so more research is needed to confirm generalizability of these results. Still, as an exploratory analysis, these findings suggest promising areas to focus future studies and interventions. Another limitation of our data was our inability to analyze the contribution of provider-level differences to variation in quality, due to missingness and small sample sizes at the provider level. Additional limitations include the lack of data collection about Indigenous identity, which prevented any analysis of how quality is experienced for a group with known health disparities and reported lower quality of care in other studies [26, 34]. Similarly, low numbers of LGBTTTIQ-identifying individuals reduced our power to detect differing experiences of quality for this group, though the low numbers themselves may represent a need to improve access. Courtesy bias may have also caused an underestimation of effects found in this study, as clients are less likely to report negative experiences [35]. This would suggest that the findings in this study may be more significant than they appear.

Despite these limitations, the overall strength of the QCC Scale is its comprehensive and nuanced breakdown of contraceptive counseling quality and its grounding in human rights-based frameworks. This or similar scales should continue to be used when evaluating differences in quality that groups of clients may be experiencing, and when exploring interventions to improve clients’ experience and contraceptive services.

## Conclusions

Significant findings from this exploratory analysis draw attention to areas where further study and interventions are needed to improve the quality of contraceptive counseling provided in Mexico. In particular, these results call for greater attention to disparities in the quality of contraceptive counseling experienced by people in different age groups, suggesting a need to investigate and prevent experiences of disrespect and abuse among younger clients and to provide more relevant information to older clients. Additionally, the quality of contraceptive counseling in different types of visits should be explored, and interventions to improve the provision of contraceptive counseling in post-partum visits are needed.

## Data Availability

The dataset supporting the conclusions of this article will be available in the repository Dryad under https://doi.org/10.7272/Q6MK6B4Q.

## Abbreviations

ICC: Intraclass correlation
LGBTTTIQ: Lesbian, gay, bisexual, transgendered, transexual, two-spirited, intersexed, queer
QCC: Quality of contraceptive counseling

## Footnotes

1 To be inclusive of the gender spectrum and identities of people who may seek to use contraceptive services, this paper avoids the term “women” and instead will use “individuals” or “clients” to refer to those people seeking contraceptive services.
